# Four-Class Classification of Neuropsychiatric Disorders by Use of Functional Near-Infrared Spectroscopy Derived Biomarkers

**DOI:** 10.3390/s22145407

**Published:** 2022-07-20

**Authors:** Sinem Burcu Erdoğan, Gülnaz Yükselen

**Affiliations:** Department of Biomedical Engineering, Acıbadem Mehmet Ali Aydınlar University, Istanbul 34684, Turkey; gulnaz.yukselen@acibadem.edu.tr

**Keywords:** fNIRS, BCI, classification, schizophrenia, obsessive compulsive disorder, migraine, Stroop test

## Abstract

Diagnosis of most neuropsychiatric disorders relies on subjective measures, which makes the reliability of final clinical decisions questionable. The aim of this study was to propose a machine learning-based classification approach for objective diagnosis of three disorders of neuropsychiatric or neurological origin with functional near-infrared spectroscopy (fNIRS) derived biomarkers. Thirteen healthy adolescents and sixty-seven patients who were clinically diagnosed with migraine, obsessive compulsive disorder, or schizophrenia performed a Stroop task, while prefrontal cortex hemodynamics were monitored with fNIRS. Hemodynamic and cognitive features were extracted for training three supervised learning algorithms (naïve bayes (NB), linear discriminant analysis (LDA), and support vector machines (SVM)). The performance of each algorithm in correctly predicting the class of each participant across the four classes was tested with ten runs of a ten-fold cross-validation procedure. All algorithms achieved four-class classification performances with accuracies above 81% and specificities above 94%. SVM had the highest performance in terms of accuracy (85.1 ± 1.77%), sensitivity (84 ± 1.7%), specificity (95 ± 0.5%), precision (86 ± 1.6%), and F1-score (85 ± 1.7%). fNIRS-derived features have no subjective report bias when used for automated classification purposes. The presented methodology might have significant potential for assisting in the objective diagnosis of neuropsychiatric disorders associated with frontal lobe dysfunction.

## 1. Introduction

In clinical practice, the majority of neuropsychiatric disorders are diagnosed with a clinician-dependent interpretation of patient information, which is obtained through a variety of subjectively biased sources such as clinical interviews, self-reports, observational data, and behavioral measures [1,2,3,4,5]. The potential of introducing subjectivity during both interpretation and acquisition of these diagnostic measures may have a prominent impact on the final clinical decision and highlights the critical need for developing accurate, objective, and reliable clinical decision support systems. Such decision support systems should ideally analyze objective and quantitative measures of the distinct characteristics of the neurobiological changes that are gradually induced by each neuropsychiatric disorder [6,7].

Within this context, various functional brain imaging modalities, such as functional magnetic resonance imaging (fMRI), positron emission tomography (PET), electroencephalography (EEG), and functional near-infrared spectroscopy (fNIRS), have been utilized for characterizing the neurobiological underpinnings of a variety of neuropsychiatric disorders [8,9,10]. Among these modalities, fNIRS systems have stepped forward for extracting informative, neuronally induced hemodynamic markers of cognition during altered brain states in naturalistic settings [11,12]. Consequently, fNIRS systems have also received increasing interest in the field of psychiatry for assisting diagnosis, prognosis, and follow-up of treatment procedures thanks to their: (1) portability, (2) non-invasive nature, (3) modest equipment size, (4) robustness to electrogenic or motion artifacts, (5) low operating cost, (6) quick set-up time and calibration, (7) ability to collect biological information at any desired frequency and duration, and (8) ease of application in ecologically valid settings to a broad range of patient populations involving children and elderly adults [13].

Indeed, recent studies have presented compelling evidence that a wide variety of neuropsychiatric disorders can be characterized by functional alterations in the hemodynamic activity of the prefrontal cortex (PFC), which can be detected with fNIRS [13]. For instance, hypoactivation in frontal lobe regions has been detected in patients with schizophrenia (SCZ) and major depressive disorder (MDD) during verbal fluency tasks when compared to their healthy counterparts [5,14]. Similarly, hyper- and hypo-connectivity between different brain regions during resting state have been identified in patients with schizophrenia (SCZ) [15,16,17,18,19] and major depressive disorder (MDD) [8,9,14], while decreased cerebral blood flow in bilateral symmetric regions of the inferior PFC has been detected in patients with obsessive compulsive disorder (OCD) when compared to their healthy counterparts [20,21]. PFC dysfunction in the form of hypo- or hyper-connectivity during resting state or hypoactivation during various cognitive tests (e.g., Stroop and verbal fluency test) has been extensively observed and reported in patient groups diagnosed with a variety of major neuropsychiatric disorders, which include SCZ, MDD, bipolar disorder (BD), post-traumatic stress disorder (PTSD), and attention deficiency and hyperactivity disorder (ADHD). Results from meta-analysis studies indicated that the topographical distributions of functional abnormalities observed in these patient groups are likely to have disorder-specific patterns [14]. Overall, these studies have highlighted the potential of exploring PFC-based neurofunctional features as objective and distinctive biomarkers of various major neuropsychiatric disorder states. They also showed that information from practical and preferably field-deployable cerebral physiology monitoring tools such as fNIRS systems can quantify and parameterize abnormalities in frontal lobe function and may have a great potential for assisting in the objective diagnosis and classification of major psychiatric disorders which, in most cases, have overlapping behavioral symptoms across each other and are difficult to distinguish when decisions are based solely on observation, self-report, interview, and/or rating scales.

Considering the critical demand to integrate more objective measures of neurophysiological alterations into diagnostic clinical decision processes, the presented study aimed to assess the feasibility and applicability of an fNIRS-based automated classification approach for accurate prediction and objective identification of the presence of three distinct neuropsychiatric or neurological disorder states which are known to induce alterations in frontal lobe function. In our recent work, we demonstrated the feasibility and applicability of an fNIRS-assisted automated classification approach for accurate prediction of the presence of impulsivity in adolescents [22]. More specifically, our results suggested that training computationally efficient supervised learning algorithms with informative features obtained from clinical, behavioral, and fNIRS-derived hemodynamic measures could serve as a decision support system for recognizing the presence of impulsivity in individuals. However, the clinical features included in the feature sets still had the potential to present subjective bias when used for algorithm training purposes because there always existed some probability that the subjects could provide false reports in the clinical interviews.

Based on the promising performance of integrating fNIRS-derived features and clinical features for recognizing the presence of impulsivity in our recent work, the objective of this study was to introduce a more reliable, machine learning-based classification approach for correct identification of the presence of three distinct neuropsychiatric and/or neurological disorder states. The proposed machine learning-based classification approach involved training three supervised learning algorithms with (i) fNIRS-derived informative biomarkers only and (ii) a combination of fNIRS-derived biomarkers and performance measures obtained during a cognitive test, named the Stroop task. We tested the feasibility of the proposed approach with three distinct supervised learning algorithms and by extending our classification problem to include four classes of subjects. The ultimate goal was to demonstrate the feasibility of an fNIRS-based automated classification methodology for predicting the presence of a neuropsychiatric disease, where input features are of pure biological origin and can be derived non-invasively in naturalistic settings by use of ergonomic fNIRS probes. For this purpose, hemodynamic information obtained from concurrent fNIRS recordings during a Stroop task was processed to extract global efficiency metrics which are indicative of the strength of functional connectivity among different PFC regions. The efficacy of training three distinct supervised machine learning algorithms, namely naive Bayes (NB), linear discriminant analysis (LDA), and support vector machines (SVM), with (a) fNIRS-derived neuronally induced biomarkers and (b) a combination of fNIRS-derived biomarkers and cognitive performance measures obtained during the Stroop task, was evaluated. The performance metrics of possible combinations of each classification algorithm and feature set combination were assessed by whether each subject was correctly labeled among the four classes, which included healthy controls (HC), patients diagnosed with migraine without aura (MIG), schizophrenia (SCZ), and obsessive compulsive disorder (OCD).

Our study presents the following novelties with respect to the current literature: To date, there have been no studies that attempted to identify the presence of a neuropsychiatric or neurological disorder by use of a four-class automated classification scheme based on a combination of fNIRS-derived neuronally induced metrics obtained during a neuropsychological test and supervised machine learning methods. The efficacy of an automated classification approach which aims to correctly label a neuropsychiatric disorder into one of four categories has not been evaluated before with structural or functional neuroimaging measures. The efficacy of combining fNIRS-derived global efficiency metrics of the PFC as sole informative features of a neuropsychiatric and/or neurological disorder with supervised learning methods has also not been evaluated before.

## 2. Materials and Methods

### 2.1. Subjects

In this study, 13 healthy control (HC) subjects (6 female (F), mean age 26), 20 migraine (MIG) patients without aura (12 F, mean age 27), 26 patients with obsessive compulsive disorder (OCD) (11 F, mean age 29), and 21 schizophrenia (SCZ) patients (10 F, mean age 28) participated. Each subject provided informed consent before participating in the experiment. The study protocol was approved by the Ethics Committee of Pamukkale University, Denizli, Turkey. All experiments were conducted according to the latest Declaration of Helsinki. Parts of these datasets have been utilized in previous works performed by our group and coworkers [23,24,25,26,27,28,29].

### 2.2. Experimental Protocol

**Figure 1:**
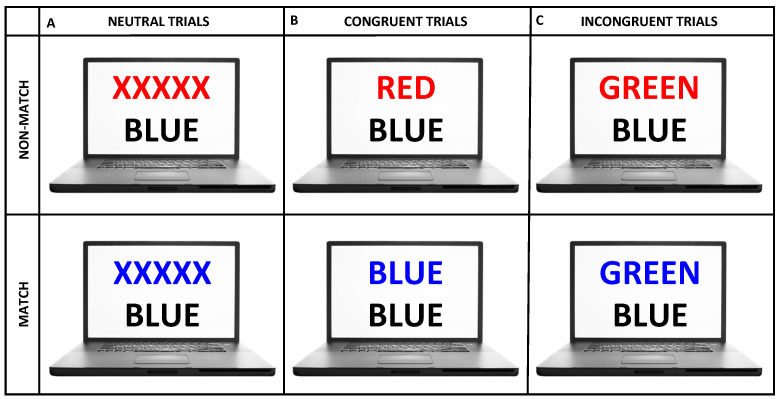
Types of stimuli that were presented within the color–word Stroop experiment. Samples of trial presentations are schematically represented for match (top row) and non-match conditions (bottom row) of (**A**) neutral, (**B**) congruent, and (**C**) incongruent stimuli.

**Figure 2:**
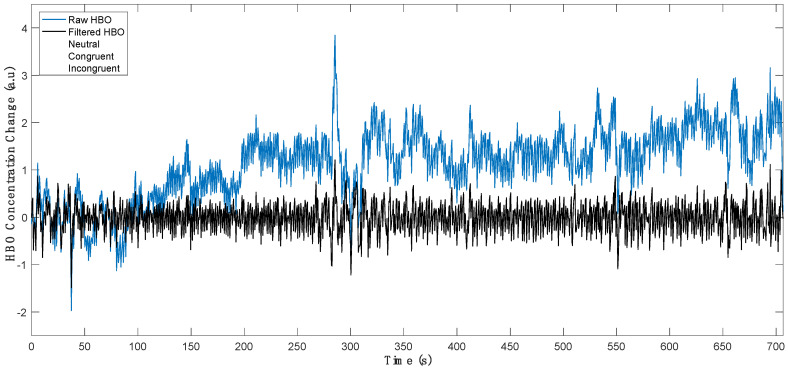
Experimental protocol depicting stimuli timing for neutral, congruent, and incongruent stimuli blocks in a sample session. HBO time series from a representative channel of a subject are plotted before (black) and after an 8th order Butterworth high-pass filter is applied with a cut-off frequency of 0.009 Hz (blue). a.u: arbitrary units.

During the experiments, subjects sat on a comfortable chair in front of a computer screen which was placed approximately 1 m away from their eyes. All experiments were carried out in a dimly illuminated, silent room. The experimental protocol was briefly explained to each subject prior to the onset of each experiment. They were requested to sit relaxed and refrain from moving their head during the fNIRS recordings. During the experiment, their task was to carefully complete a color–word Stroop task which was adapted to Turkish from a pioneer protocol proposed by Zysset et al. [30]. Each experiment began with 30 s of a baseline recording followed by presentation of alternating blocks of 3 stimulus conditions which consisted of neutral (N), congruent (C), and incongruent (IC) stimuli (Figure 1 and Figure 2). There was a total of 5 stimulus blocks for each condition (i.e., N, C, IC) and all task blocks were presented in a randomized order that changed for every experimental session. Each stimulus block consisted of 6 different trials of the same condition. Within a block, each trial appeared on the screen for 2.5 s followed by a 4 s blank screen. Task blocks were separated with 20 s periods of rest (Figure 2).

During each stimulus presentation, two rows of letters were displayed on the screen. The task was to evaluate whether the color of the letters displayed at the top row matched with the meaning of the word displayed at the bottom row. Subjects were asked to press the left button of the mouse if the color of the upper row letters matched with the meaning of the bottom row word. These cases were called match cases (Figure 1, top panel). They were asked to press the right button if the color of the upper row letters did not match with the meaning of the bottom row word for non-match cases (Figure 1, bottom panel). The letters in all trials were printed in one of four basic colors, which were yellow, red, blue, or green. In N trials, top row letters were written in yellow, red, blue, or green but did not form a meaningful word, and a color name was typed in black on the bottom row. For C trials, a word with the meaning of a color was typed in the same congruent color in the top row. For the IC trials, a word with the meaning of a color was typed in another color (i.e., incongruent) in the top row. Hence, subjects had to suppress processing the color information and evaluate the meaning information of the top row letters while making a comparison with the meaning information of the bottom row letters to provide a correct answer for the IC trials. Such an interference between two competing cognitive inputs induced a Stroop effect [23,24,25,26,27,30]. The number of match and non-match cases for each trial type was balanced during the experiment. The average reaction time and error rate were calculated for N, IC, and C trials separately.

### 2.3. fNIRS Data Acquisition

**Figure 3:**
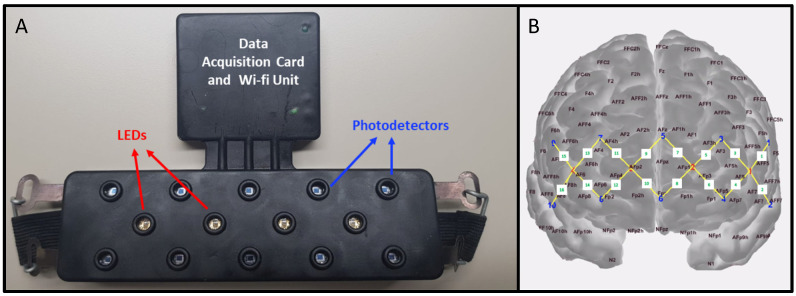
(**A**) Configuration of the forehead probe and (**B**) approximate location of the LEDs and photodetectors with respect to the international 10–20 system for electrode placement. LEDs are demonstrated with red dots and detectors are represented with blue dots. Yellow lines are drawn between the LED–photodetector pairs, which form a total of 16 channels.

Hemodynamic signals were collected from the prefrontal cortex region with a wireless ARGES-CEREBRO system (Hemosoft Information Technology and Training Services Inc., Ankara, Turkey) [24,25,26,31,32,33] which has a flexible forehead probe equipped with 4 light emitting diodes (LEDs) and 10 photodetectors (Figure 3A). The LED–photodetector pairs with 2.5 cm distance were accepted as channels and a total of 16 equidistant channels were formed which covered parts medial, orbitofrontal, and dorsolateral cortices (Figure 3B). Each LED emits near-infrared light at 750 and 850 nm in continuous wave mode and the sampling rate of the system is 1.77 Hz. The ability of this probe design to allow light penetration through the cortical tissue and collect hemodynamic information from the anterior part of the PFC has been discussed extensively in previous work by our group [23,33]. Wavelength-specific light intensity changes were detected at each detector separately, and this information was converted to optical density (OD) changes of each wavelength for each channel. Channels whose raw light intensity signals presented coefficient of variability (C.V) above 7.5% (C.V = 100 × standard deviation(signal)/mean(signal)) were not included in the analyses [34]. Time series of OD changes were provided as inputs to the modified Beer–Lambert law to compute channel-specific changes in localized HBO and HBR concentrations [10,11,35,36]. The partial pathlength factor was taken as 6 for both wavelengths [37,38,39]. HBO signals were visually inspected to exclude trial blocks which had motion artifacts within a time window spanning 5 s pre- and post-stimulus duration. Changes in HBO concentration have been reported to be a better indicator of alterations in neuronal metabolism induced by cognitive tasks [40,41,42,43,44], while having a higher signal-to-noise ratio when compared to HBR signals [40,41,44]. Hence, the efficacy of only HBO-derived hemodynamic features was tested for classification purposes.

### 2.4. Data Analysis

#### 2.4.1. Processing of fNIRS Signals

The fNIRS-HBO signals are composed of neuronally and systemically induced hemodynamic components which are intermixed with each other over a broad range of frequencies. The neuronally induced hemodynamic variations in the HBO signal are caused by both spontaneous and task-related neuronal activity, while the systemic physiological activity-related hemodynamic components have multiple origins, which include variations in heartbeat, respiration, blood pressure, and vascular tone. Hence, prior to obtaining correlation-based functional connectivity metrics between HBO signals of different channel pairs, the impact of common, global systemic effects of non-neuronal origin inherent in both channel data had to be reduced. Such a procedure is necessary to isolate the extent of correlation caused by only neuronally induced hemodynamic effects, since common physiological effects of non-neuronal origin could inflate the correlation between signals of channel pairs. A partial correlation approach was adapted from the works of Akin [28] and Akin [29] to reduce the impact of common systemic interference to Pearson’s correlation coefficients calculated between HBO signals of each channel pair. Similar to these works, HBO signals of all channels were initially high-pass-filtered with a cut-off frequency of 0.009 Hz using an 8th order Butterworth filter. The high-pass-filtered HBO signals were then averaged to have a single global signal regressor, which was utilized as the partial regressor for modeling and removing the impact of common systemic noise from the correlations between each channel pair in the subsequent step of the analysis [22,24,25,26,27,28,29].

Time traces used for computing the correlation between each channel pair were obtained as follows. For each channel, HBO signals corresponding to each stimulus block were truncated from the onset to the end of that block. These time segments were then concatenated in time to obtain a single task-related HBO signal for each channel of each subject. Similarly, the partial correlation regressor was obtained by truncating and concatenating the time segments belonging to all task blocks in the global signal regressor. Then, 16-by-16 partial correlation (PC)-corrected functional connectivity (FC) matrices for each subject were generated after removing the impact of this partial correlation regressor [22,29].

#### 2.4.2. Computation of Cognitive Quotient and Global Efficiency Features

Two groups of features were extracted from the behavioral and hemodynamic data obtained during the Stroop task. Similar to our previous work [22], the behavioral performance was quantified with a feature named the cognitive quotient (CQ), which could be considered as a generalized cognitive performance indicator of each subject during the Stroop task. The accuracy and reaction time metrics obtained from all trials of the Stroop experiment were fused in this single metric by dividing the overall accuracy performance (i.e., percentage of correct answers over all trials) with the average reaction time for all trials.

Regarding the hemodynamic features, a relatively novel functional connectivity metric called global efficiency (GE) was obtained from the 16-by-16 partial correlation-corrected FC matrices obtained for each subject. The GE metric was obtained from a graph theoretical network analysis approach, and its efficacy in demonstrating the degree of connectedness and information transfer between cortical regions during various cognitive tasks has been shown in previous studies [22,27,28,29].

**Figure 4:**
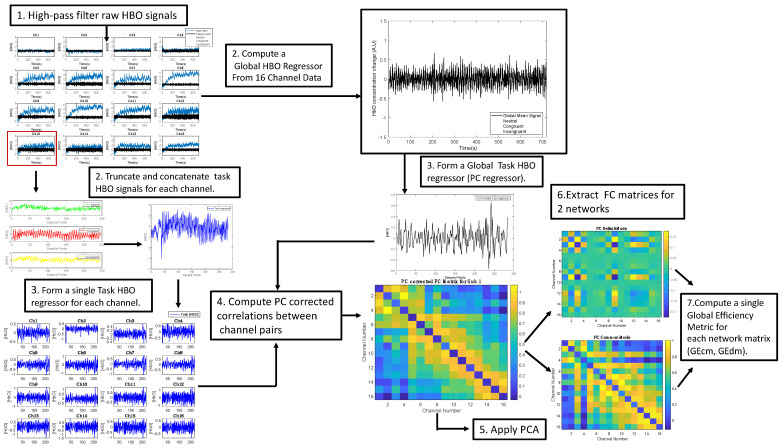
Pipeline for extracting fNIRS derived features.

After the partial correlation-corrected FC matrices were obtained for each subject, these matrices were decomposed into two matrices, which represent the degree of connectedness of the default mode (DM) and the cognitive mode (CM) networks of the brain. This decomposition was established by applying principal component analysis to the FC matrix, the details of which are extensively explained in the recent work of Akin [29]. Briefly, principal component (PC) decomposition was applied to the 16 by 16 FC matrices and the weights of the PCs were thresholded using an optimization procedure described in [29]. The DM and CM components of the FC matrices were reconstructed by weighting and summing the PC regressors that had weights below and above the threshold, separately. GE values of the DM and the CM components were computed separately for each subject by using the formula of Latora and Marchiori [45]. The GE feature for the DM network was named GE_dm_, and similarly, the GE feature for the CM was named GE_cm_ (Figure 4).

#### 2.4.3. Classification Methods

The feasibility of training fNIRS-derived GE features with machine learning classifiers for correct identification of the presence of a disorder in each subject was evaluated and compared for three distinct algorithms. These algorithms were naive Bayes (NB), linear discriminant analysis (LDA), and support vector machines (SVM). These algorithms were selected for several reasons: (1) They have been shown to perform well with small sample sizes (n < 200) [5,15,22,46,47,48,49,50,51,52,53,54,55,56,57]. (2) Their computational cost is low. (3) They have performed successfully in a variety of classification problems where fNIRS features extracted during cognitive and motor tasks were utilized [25,26,28,29,30,31]. (4) Their good performance for classification at the single subject level has been reported for previous neuropsychiatry studies with similar sample sizes, but a lower number of classes [1,5,15,58,59,60,61,62]. The mathematical architecture of these algorithms has been extensively explained in previous work performed by our group [22,46] and others [52,53,54,55,56].

**Figure 5:**
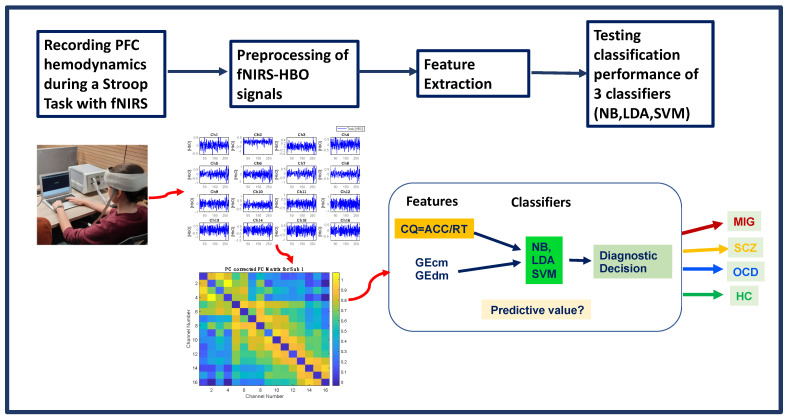
Flowchart of the system design.

Each classification algorithm was constructed by using the libraries of the WEKA platform (version 3.8.5) [63]. The sequential minimal optimization (SMO) algorithm was utilized for training the SVM classifier [64]. SMO was run with the Pearson VII universal kernel [65], also known as the PUK kernel. To avoid overfitting, the regularization parameter (C) of SMO and PUK kernel parameters (i.e., omega (ω) and sigma (σ)) was optimized by maximizing the accuracy with a grid-search procedure. Assigning C = 10 and ω = σ = 1 yielded the best results. LDA and NB classifiers were constructed with the default parameters implemented in the WEKA software. A brief flowchart of the processing pipeline is demonstrated in Figure 5.

#### 2.4.4. Performance Evaluation

To evaluate and compare the classification performances of each algorithm, accuracy, precision, sensitivity, specificity, and F1-score were calculated through a comparison of the actual and predicted labels of test data [61]. For each algorithm, performance metrics were obtained after 10 runs of a 10-fold cross-validation (C.V) procedure, where in each run, 1/10th of the subject data were separated for testing the algorithm and the remainder of subject data were used for training, and this procedure was repeated 10 times. For each performance metric, the mean scores across all runs and their standard deviation were computed (Table 1 and Table 2). This procedure was conducted for cases when each algorithm was trained with (i) fNIRS only features (i.e., GE_cm_ and GE_dm_) and (ii) a combination of fNIRS-derived features (i.e., GE_cm_, GE_dm_) and a behavioral feature (i.e., CQ). All features were computed for each subject separately.

Pairwise comparisons between the performance metrics obtained from each possible algorithm (i.e., NB, LDA, SVM) and feature set (i.e., GE_cm_ + GE_dm_ or GE_cm_ + GE_dm_ + CQ) combination were performed with two-tailed, two-sample *t*-tests. Comparisons of each performance metric (i.e., accuracy, precision, recall, specificity, and F1-score) among different combinations of algorithm and feature set choices aimed to assess: (i) whether training each algorithm with only fNIRS features resulted in a statistically significantly different classification performance when compared to training the same algorithm with a combination of fNIRS and behavioral features, and (ii) whether there exists an algorithm and feature set combination with a statistically significantly higher performance when compared to all other options.

## 3. Results

**Figure 6:**
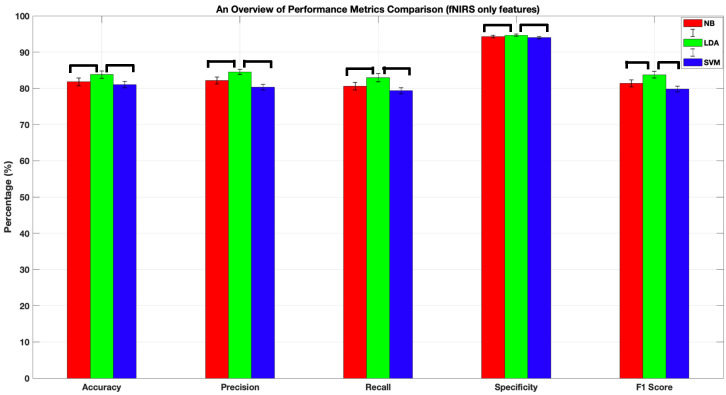
Classification performances of NB, LDA, and SVM algorithms after being trained with fNIRS-derived features (i.e., GE_cm_ and GE_dm_). Horizontal lines depict statistically significant differences between performances of different algorithm pairs. All algorithms achieved accuracy, precision, recall, and F1-score performances above 80%, while the specificity scores were above 94%. LDA performed significantly higher than both SVM and NB in terms of accuracy, precision, recall, specificity, and F1-score. The error bars represent standard error of the mean performance after 10 runs of a 10-fold C.V.

Table 1 presents the four-class classification performances of NB, LDA, and SVM classifiers when they were trained with two fNIRS-derived features (i.e., GE_cm_, GE_dm_). All algorithms achieved accuracy, precision, recall, and F1-score performances above 81%, while the specificity scores were all above 94%. It should be noted that LDA performed significantly higher than both SVM and NB (Figure 6) in terms of accuracy (83.8 ± 1%, *p* < 0.05), precision (85 ± 0.01%, *p* < 0.05), recall (83 ± 0.01%, *p* < 0.05), specificity (95 ± 0.01%, *p* < 0.05), and F1-score (84 ± 0.01%, *p* < 0.05). The performances of NB and SVM were not statistically significantly different in terms of the reported metrics.

**Figure 7:**
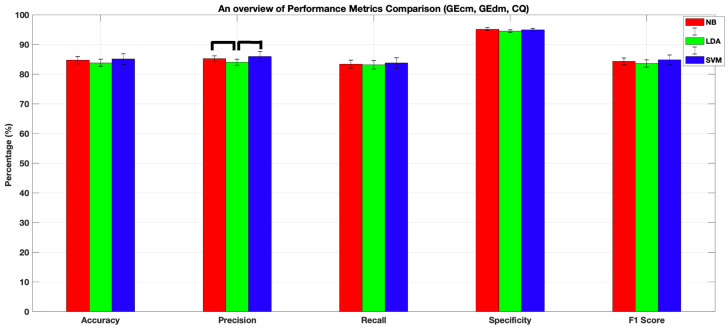
Classification performances of NB, LDA, and SVM algorithms after being trained with a combination of fNIRS and behavioral features (i.e., GE_cm_, GE_dm_, and CQ). Horizontal lines depict statistically significant differences between performances of different algorithm pairs. All algorithms achieved accuracy, sensitivity, precision, and recall performances above 83%, while the specificity scores were all above 94%. There was no statistically significant difference among accuracy, recall, specificity, and F1-score performances of the three algorithms. The error bars represent standard error of the mean performance after 10 runs of a 10-fold C.V.

Table 2 presents the four-class classification performances of NB, LDA, and SVM classifiers when they were trained with fNIRS and behavioral features (i.e., GE_cm_, GE_dm_, and CQ). Comparisons between the performance of each tabulated algorithm with respect to the corresponding performance obtained with fNIRS only features (Table 1) were performed with paired *t*-tests, and bold-typed results (Table 2) denote significantly higher performance of the corresponding algorithm when compared to the results when the same algorithm is fed with fNIRS only features. All algorithms achieved accuracy, precision, recall, and F1-score performances above 83%, while the specificity scores were all above 94%. Feeding NB and SVM with a combination of fNIRS and behavioral features resulted in a statistically significantly higher performance in each metric when compared to the performance obtained by training the same algorithm with fNIRS only features. However, LDA achieved a similar performance in each metric regardless of the type of feature set combination utilized for training. There were no statistically significant differences in accuracy, recall, specificity, and F1-scores among the three algorithms. Nonetheless, the precision score obtained with SVM was statistically significantly higher than both LDA and NB (86 ± 1.6%, *p* < 0.05, Table 2 and Figure 7).

Training LDA with fNIRS only features resulted in a comparable performance with the performance metrics obtained when the same algorithm was trained with a combination of fNIRS and behavioral features. A statistical comparison of the performance of the best-performing algorithm (LDA) and fNIRS only feature set combination of Table 1 with the performance metrics of NB and SVM of Table 2 demonstrated that no significant difference existed between any algorithm pair for accuracy, recall, specificity, and F1-scores.

To sum up, we conclude that training LDA with fNIRS only features results in a comparable performance with training the three supervised algorithms with a combination of fNIRS and behavioral features. Regarding the best performance, although there were no statistically significant differences among the three algorithms for accuracy, recall, specificity, and F1-scores (Figure 6), we should still note that SVM had the best performance in all metrics when trained with a combination of fNIRS and behavioral features obtained during the Stroop task (Table 1 and Table 2 and Figure 7).

**Figure 8:**
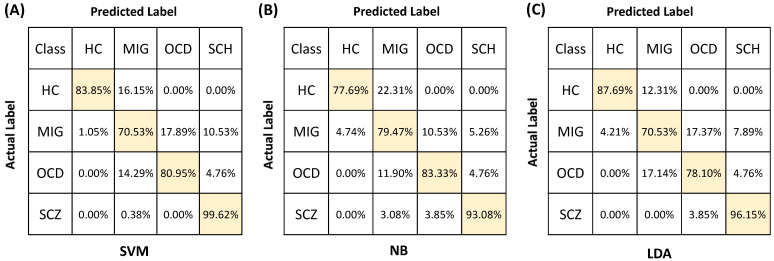
Confusion matrices depicting the true-positive (shaded in yellow) and false-negative predictions of (**A**) SVM, (**B**) NB, (**C**) LDA when they are trained with a combination of fNIRS and behavioral features.

Figure 8 presents the confusion matrices for each algorithm, which demonstrate the true-positive and false-negative predictions attributed to each class. All algorithms achieved classification accuracies above 70% for each class. All algorithms demonstrated the highest true-positive prediction rate for SCZ patients, which was followed by OCD, HC, and MIG. SCZ and OCD subjects were not misclassified as HCs for any of the algorithms. This result is significant as these two patient groups are expected to have the most distinct alterations in cognitive performance and cerebral hemodynamic activity during the Stroop task when compared to the HC group [13,17,66,67,68,69,70,71]. The fact that HC subjects were not misclassified as OCD or SCZ for any of the algorithms suggests the distinctive and physiology-related informative power of the selected features. However, HC subjects could be falsely attributed to the MIG class (SVM: 1.05%, NB: 4.74%, LDA: 4.21%) regardless of the algorithm type. This result is not surprising as MIG subjects were tested during the interictal period while they were exempt from attacks, hence their cognitive performance and the relevant spatial and topographic distribution of functional activation might have been similar to HCs during the interictal period. The consistencies in the classification performance patterns of the three algorithms as well as the consistency of performance results with physiology-related information highlight the distinctive power and biologically informative nature of the fNIRS-derived features utilized in the study. It can be concluded that training NB, LDA, and SVM with fNIRS-derived metrics demonstrates a differential diagnosis potential, regardless of the mathematical architecture of the algorithm.

## 4. Discussion

The current diagnostic model for a majority of neuropsychiatric disorders relies on evaluation of measures which include clinical, observational, and/or behavioral scales that are obtained through interviews, questionnaires, observations, self-reports, and/or neuropsychiatric test batteries [3,4,5]. However, subjectivity introduced during both collection and clinical interpretation of these multi-domain measures brings forth the demand for more objective diagnostic markers. The high variability in clinical decisions for similar cases observed across different clinicians, cultures, and countries highlights the critical need for developing more objective decision support systems for diagnosis, which should ideally be based on quantitative measures of the neurophysiological alterations underlying each disorder.

Taking this critical demand into consideration, the presented study aimed to assess the feasibility and applicability of an fNIRS-based automated classification approach for accurate prediction and objective identification of the presence of three distinct neuropsychiatric or neurological disorder states which are known to induce alterations in frontal lobe function. The proposed machine learning-based classification approach involved training various supervised learning algorithms with (i) novel fNIRS-derived informative biomarkers and (ii) a combination of fNIRS-derived biomarkers and performance measures obtained during a neuro-cognitive test, named the Stroop task. We tested and compared the efficacy of training three commonly employed and computationally efficient supervised learning algorithms with these neuronally induced biomarkers, and their comparably high performances were demonstrated with accuracy, precision, recall, specificity, and F1-scores. The performance of each algorithm in the correct identification of the presence of a disorder in each subject was evaluated by whether the subject was correctly labeled among the four classes, which included HCs, MIG, SCZ, and OCD. Hence, four-class brain–computer interface system designs were formulated which simply included the collection of hemodynamic signals with an fNIRS system while the subject was engaged in a Stroop task. Two global efficiency features were obtained from the PFC HBO signals, and accuracy and reaction rate performance obtained during the Stroop task were fused in a single behavioral feature, named the cognitive quotient (CQ). The comparably high performance scores obtained with the three classification algorithms, which have distinct mathematical architectures, highlighted the informative nature of these neuronally induced features. They also demonstrated the promising nature of integrating fNIRS-derived features together with cognitive performance scores from neuropsychiatric test measures and multivariate pattern analysis (MVPA) approaches for accurate recognition of neuropsychiatric disorder states. Our methodological approach resulted in increased classification accuracy when compared to the brain–computer interface (BCI) study designs conducted with fNIRS for other classification purposes, such as decoding mental thought processes or motor imagery signals [71,72].

In the following sections, we first evaluate the efficacy of NB, LDA, and SVM in correct identification of the presence of a disorder at the single subject level and we discuss the differential diagnostic potential of the proposed approach. We then highlight the importance of our findings, discuss the limitations of our study, and propose recommendations for future work.

### 4.1. Comparison of the Classification Performances of LDA, NB, and SVM

Training NB, LDA, and SVM with two fNIRS-derived functional connectivity metrics resulted in accuracy, precision, recall, and F1-score performances above 81%, while the specificity scores were all above 94%. While the performance metrics obtained with each algorithm had a very close range, it should be noted that LDA performed significantly higher than both SVM and NB in terms of accuracy (83.8 ± 1%, *p* < 0.05), precision (85 ± 0.01%, *p* < 0.05), recall (83 ± 0.01%, *p* < 0.05), specificity (95 ± 0.01%, *p* < 0.05), and F1-score (84 ± 0.01%, *p* < 0.05) when trained with fNIRS only features. A statistical comparison of the performance of the best-performing algorithm (LDA) and fNIRS only feature set combination of Table 1 with the performance metrics obtained by training each algorithm with a combination of fNIRS and behavioral features demonstrated that no significant difference existed between the performances of any algorithm pair for accuracy, recall, specificity, and F1-scores. Hence, we conclude that training LDA with fNIRS only features results in a comparable performance with training the three supervised algorithms with a combination of fNIRS and behavioral metrics. Regarding the best performance, we should note that SVM had the best performance in all metrics when trained with a combination of fNIRS and behavioral features obtained during a Stroop task (Table 1 and Table 2 and Figure 6). However, we should also note that SVM did not have a statistically significantly higher performance than the rest of the algorithms for the majority of the performance metrics (i.e., accuracy, recall, specificity, and F1-scores reported in Figure 6). Hence, we can conclude that the utilized features are distinctive in nature as they performed well with all three classifiers regardless of the mathematical architecture of the algorithm. Obtaining a high classification performance with all classifiers highlights the feasibility and applicability of feeding machine learning-based methods with fNIRS-derived neuro-cognitive biomarkers for classification of disorder states associated with alterations in frontal lobe function.

With the recent advances in the computational power of daily used computers, MVPA methods have received increasing interest for automated identification and objective recognition of neurological and neuropsychiatric disorder states by use of structural and functional neuroimaging features. The majority of these studies examined the diagnostic potential of utilizing multivariate features for: (i) correct identification of the presence of a disease state, (ii) rating the severity of a clinical state, or (iii) differentiating subgroups of patients. Arabshirani et al. provided an excellent review of previous neuroimaging studies that aimed at single-subject prediction of neurological, neurodegenerative, or neuropsychiatric disorders by use of structural and functional imaging features [61], while Orru et al. presented an extensive summary of the previous studies that utilized SVM for differentiating a neuropsychiatric disease state from a healthy state [73]. Regarding automated recognition of SCZ, Steardo et al. provided a review of classification studies that utilized a combination of SVM and neuroimaging markers [58]. The majority of these studies reported binary classification performances for differentiating a disorder state from a healthy state and the reported accuracies ranged from 67% to 100%. Regarding differentiation of OCD from a healthy state, the highest performance metrics were reported by Sen et al., who proposed the utility of resting state functional connectivity-derived network features with SVM [74]. They achieved 80% accuracy, 81% sensitivity, and 77% specificity with a relatively small sample size (n = 16 for OCD and n = 13 for HC). Similarly, three studies utilized MVPA methods and MRI-based neuroimaging markers for accurate prediction of the presence of migraine by use of two-class classification schemes, and the reported accuracies ranged between 80% and 96% [75,76,77].

Among three-class classification studies, Yu et al. reported a study where they used several MVPA methods to discriminate healthy controls (n = 38), schizophrenic patients (n = 32), and patients diagnosed with major depression disorder (n = 19). They achieved a correct classification rate of 81% using functional connectivity features from resting state fMRI scans [59]. Their sample size was also similar to our study. Kawazaki et al. built a binary classification model for differentiating SCZ from HC utilizing voxel-based morphometry features from MRI with a small dataset (n = 30 per class). Their classification accuracy performance was 80% [78]. Yassin et al. performed a three-class classification study where they trained several machine learning algorithms for accurate identification of autism spectrum disorder, healthy controls, and SCZ patients. The best results were achieved with MRI-derived cortical thickness parameters using a logistic regression (LR) classifier. Their overall maximum classification accuracy was reported as 69%. The maximum binary classification accuracies between different class pairs were less than 80% when tested with several classifiers, including SVM [79].

We should note that an objective comparison of our performance results with the performances reported in previous studies is complicated since the study designs differed in terms of sample size, number of classes, type and number of features, disorder types, C.V procedure, and the selected classifiers (Table 3). Nonetheless, we can still conclude that the performance metrics achieved with our four-class classification methodology fall in the high-performance spectrum among the performance metrics reported in previous studies, which targeted classification of various neuropsychiatric populations from healthy counterparts by use of structural and functional neuroimaging measures.

### 4.2. Potential of the Proposed Methodology for Differential Diagnosis

Comorbidities often exist among major neuropsychiatric disorders in the form of overlapping behavioral symptoms and similar neurobiological alterations. Hence, one of the major challenges for a precise diagnostic decision is to be able to differentially diagnose neuropsychiatric disorders which have overlapping symptoms, such as SCZ, MDD, and BD [14,57]. While differential diagnosis of the patient groups presented in this study would be easy to decipher at the clinical stage, we should emphasize the fact that our work serves as a proof-of-concept study to demonstrate the utility of combining fNIRS-derived functional connectivity metrics obtained during a cognitive test with machine learning-based classification methods for assisting accurate classification and objective identification of neuropsychiatric disorder states associated with frontal lobe functional abnormalities.

Recent studies have presented compelling evidence that a wide variety of neuropsychiatric disorders are characterized with alterations in the neural activity of the PFC [13]. However, whether there exists a distinct topographical distribution of functional abnormalities specific to each neuropsychiatric disorder and whether each neuropsychiatric disorder can be associated with a distinct abnormality in cerebral activation that can be recognized by fNIRS during a cognitive test remains unclear. In our study, all algorithms achieved classification accuracies above 70% for each class. All algorithms demonstrated the highest true-positive prediction rate for SCZ patients, which was followed by OCD, HC, and MIG. HC subjects were not misclassified as OCD or SCZ for any of the algorithms. These two patient groups are expected to have the most distinct alterations in cognitive performance and cerebral hemodynamic activity during the Stroop tasks when compared to the HC group [13,17,66,67,68,69,70]. Hence, the fact that HC subjects were not misclassified as OCD or SCZ for any of the algorithms suggests the distinctive and physiology-related informative power of the selected features. HC subjects could be falsely attributed to the MIG class. This result is not surprising as MIG subjects were tested during the interictal period which might be cognitively similar to a healthy state, and hence the spatial and topographic distribution of their functional activation might not be significantly different from HCs during the Stroop task. OCD and SCZ subjects were not misclassified as HCs for any of the algorithms. The consistencies in the classification performance patterns of the three algorithms as well as the consistency of performance results with physiology-related information highlight the distinctive power and biologically informative nature of the fNIRS-derived features utilized in the study.

To sum up, our results suggest that training NB, SVM, or LDA with the fNIRS-derived global efficiency metrics obtained during a Stroop task demonstrates a differential diagnosis potential, regardless of the mathematical architecture of the algorithm. Our findings also support the notion that some novel neuro-biological features obtained with fNIRS methodology during cognitive tasks might serve as distinct signatures of the spatiotemporal characteristics of different neuropsychiatric disorder states which are associated with frontal lobe function abnormalities. Exploration of such informative and biologically derived features and combining them with machine learning-based classification approaches may have significant potential for differential diagnoses of psychopathologies which have comorbidities and overlapping symptoms.

### 4.3. Limitations of the Study and Recommendations for Future Work

We should note that the sample sizes of our subject groups were still small, although they exceeded the sample sizes reported in many of the previously reported classification studies in neuropsychiatry literature [58,61,73,74,75,76,77,78,79,80]. As a continuation of this study, we will test the performance of our methodology on a larger subject cohort. Our classification problems will include a higher number of disorder types and we will test the efficacy of identifying patients with comorbidities. We will also test the informative power of extracting hemodynamic and cognitive features from concurrent fNIRS recordings taken during a variety of neuropsychological tests which target different aspects of cognition.

Deep learning (DL) techniques have a great potential to improve the performance of fNIRS-based BCI systems if sufficiently large training sets are available [81,82]. The major advantages of these techniques rely on their ability to capture the complexity of neural information embedded in the HBO signal patterns through optimization of the network structures [81]. Indeed, there exists some successfully implemented DL classifiers with fNIRS and EEG signals [82,83,84,85,86]. However, we avoided testing the utility of DL algorithms in the presented work because of the limited cohort size of each group. Models constructed with DL algorithms have a tendency to overfit data when they are trained with small sample sizes (i.e., n < 5000) [81]. Future work will involve testing the efficacy of DL algorithms for addressing the presented classification problem in a larger cohort size and by utilizing data augmentation procedures.

In the presented study, clinical diagnosis of each participant was performed by experienced psychiatrists after careful follow-up procedures, and their final clinical decision was considered the golden standard. Hence, we could test and report the performance of each algorithm by whether it could correctly predict the final clinical decision of an experienced psychiatrist whose decision is considered as ground truth. Although the participants included in the study were reported to have strong and distinct symptoms and the clinicians had good clinical expertise for making a correct diagnosis, there still exists a possibility that some of the patients might have been given a different diagnosis by a different group of clinicians and might be incorrectly labeled. Hence, we can only report the value and high performance of combining fNIRS only markers and supervised learning algorithms in correctly predicting the clinical decision of an experienced clinician. Nonetheless, such a decision support system still might assist young clinicians who have not gained enough expertise with patients.

While the differential diagnosis of the patient classes reported in this study might not be a difficult problem in the clinics, we should note that this is a proof-of-concept study for demonstrating the potential of predicting a clinical decision through analysis of informative hemodynamic features obtained noninvasively in a clinical setting with a wearable and ergonomic fNIRS system design. Hemodynamic information can be collected with similar system designs during similar cognitive tests or vasomechanical challenges and can be processed to extract biomarkers which can be used for differential diagnosis of neurological or neuropsychiatric disorders that are known to induce abnormalities in PFC function.

## 5. Conclusions

The overarching goal of this study was to test the feasibility of an fNIRS-based BCI system design for accurate and objective identification of the presence of neuropsychiatric or neurological disorders. Our results demonstrate the potential of training supervised learning algorithms with fNIRS-derived hemodynamic and cognitive features for precise recognition of the presence of a neurological or neuropsychiatric disorder at the single-subject level. They also highlight the promise of exploring PFC-based neurofunctional features as distinctive and objective biomarkers of neuropsychiatric or neurological disorders which are associated with alterations in frontal lobe function. Neuronally induced biomarkers can be easily obtained in clinical settings with portable, wearable fNIRS systems. Such system designs might also have great potential for objective classification and differential diagnosis of major neuropsychiatric disorders which, in most cases, have overlapping behavioral symptoms across each other and are hard to distinguish when decisions are based solely on observation, self-report, interview, and/or rating scales.

## Figures and Tables

**Table 1:** Four-class classification performances of NB, LDA, and SVM algorithms when trained with fNIRS only features (i.e., GE_cm_ and GE_dm_). Each performance metric is represented in percentages (%) as the mean value across all runs ± standard deviation of the mean.

Method	Accuracy	Precision	Recall	Specificity	F1-Score
**NB**	81.77 ± 1.06	82.1 ± 1	81 ± 0.01	94 ± 0.004	81 ± 1
**LDA**	83.8 ± 1	85 ± 0.01	83 ± 0.01	95 ± 0.01	84 ± 0.01
**SVM**	81 ± 0.84	80 ± 0.01	79 ± 0.01	94 ± 0.003	80 ± 0.008

**Table 2:** Four-class classification performances of NB, LDA, and SVM when trained with fNIRS and behavioral features (i.e., GE_cm_, GE_dm_, and CQ). Each performance metric is represented in percentages (%) as the mean value across all runs ± standard deviation of the mean. Bold-typed results denote significantly higher performance of the corresponding algorithm with respect to the results when the algorithm is fed with fNIRS only features.

Method	Accuracy	Precision	Recall	Specificity	F1-Score
**NB**	**84.68** **±** **1.3**	**85** **±** **0.01**	**83** **±** **0.01**	**95** **±** **0.01**	**84** **±** **0.01**
**LDA**	83.8 ± 1.6	84 ± 1.1	83 ± 1.4	94 ± 0.04	84 ± 1.2
**SVM**	**85** **±** **1.77**	**86** **±** **1.6**	**84** **±** **1.7**	**95** **±** **0.5**	**85** **±** **1.7**

**Table 3:** Comparison of the classification performances of the discussed studies.

Author/s (Year)	Sample Size	Classifier(s)	Number of Classes	Features	Mean Accuracy (%)
Sen et al. (2017) [74]	16 OCD, 13 HC	SVM	2	Resting state network features derived from fMRI data	80
Chong et al. (2016) [75]	58 MIG, 50 HC	Quadratic Discriminate Analysis	2	Resting state network features derived from fMRI data	86
Yang et al. (2018) [76]	21 MIG without aura, 15 MIG with aura, 28 HC	Convolutional Neural Networks	2 and 3	Resting state network features derived from fMRI data	85–99 (2 class), 87(3 class)
Hernandez et al. (2014) [77]	15 HC, 20 MIG, 19 Medication Abuse	SVM	2	Graph theoretical features derived from fMRI data	87
Yu et al.(2013) [59]	32 SCZ, 19 MDD, 38 HC	SVM	3	Resting state network features derived from fMRI data	81
Kawasaki et al.(2007) [78]	30 SCZ, 30 HC	Multivariate Linear Model	2	Voxel based morphometry features extracted from MRI data	80
Yassin et al. (2020) [79]	64 SCZ, 36 ASD, 106 HC	Logistic Regression	3	Cortical thickness and subcortical volume features derived from MRI data	69
Pardo et al. (2006) [80]	10 SCZ, 10 BP, 8 HC	LDA	3	Neuropsychiatric test scores and structural metrics derived from MRI data	96
**Present work**	20 MIG, 26 OCD, 21 SCZ, 13 HC	LDA, SVM, NB	4	Cognitive quotient and Global Efficiency metrics derived from fNIRS data	84.7 (LDA), 83.8(NB), 85 (SVM)

## Data Availability

Data is available upon request.

## References

[1] Klöppel S., Abdulkadir A., Jack C.R., Koutsouleris N., Mourão-Miranda J., Vemuri P. (2012). Diagnostic neuroimaging across diseases. Neuroimage.

[2] Singh I., Rose N. (2009). Biomarkers in psychiatry. Nature.

[3] World Health Organization (1992). International Statistical Classification of Diseases.

[4] American Psychiatric Association (1994). Diagnostic and Statistical Manual of Mental Disorders.

[5] Azechi M., Iwase M., Ikezawa K., Takahashi H., Canuet L., Kurimoto R., Nakahachi T., Ishii R., Fukumoto M., Ohi K. (2010). Discriminant analysis in schizophrenia and healthy subjects using prefrontal activation during frontal lobe tasks: A near-infrared spectroscopy. Schizophr. Res..

[6] Aboraya A., Rankin E., France C., El-Missiry A., John C. (2006). The Reliability of Psychiatric Diagnosis Revisited: The Clinician’s Guide to Improve the Reliability of Psychiatric Diagnosis. Psychiatry.

[7] Ward C.H., Beck A.T., Mendelson M., Mock J.E., Erbaugh J.K. (1962). The psychiatric nomenclature. Reasons for diagnostic disagreement. Arch. Gen. Psychiatry.

[8] Drevets W.C. (2000). Neuroimaging studies of mood disorders. Biol. Psychiatry.

[9] Okada G., Okamoto Y., Yamashita H., Ueda K., Takami H., Yamawaki S. (2009). Attenuated prefrontal activation during a verbal fluency task in remitted major depression. Psychiatry Clin. Neurosci..

[10] Weyandt L., Swentosky A., Gudmundsdottir B.G. (2013). Neuroimaging and ADHD: fMRI, PET, DTI Findings, and Methodological Limitations. Dev. Neuropsychol..

[11] Ferrari M., Quaresima V. (2012). A brief review on the history of human functional near-infrared spectroscopy (fNIRS) development and fields of application. Neuroimage.

[12] Scholkmann F., Kleiser S., Metz A.J., Zimmermann R., Pavia J.M., Wolf U., Wolf M. (2014). A review on continuous wave functional near-infrared spectroscopy and imaging instrumentation and methodology. Neuroimage.

[13] Ehlis A.-C., Schneider S., Dresler T., Fallgatter A.J. (2014). Application of functional near-infrared spectroscopy in psychiatry. Neuroimage.

[14] Yeung M.K., Lin J. (2021). Probing depression, schizophrenia, and other psychiatric disorders using fNIRS and the verbal fluency test: A systematic review and meta-analysis. J. Psychiatr. Res..

[15] Song H., Chen L., Gao R., Bogdan I.I.M., Yang J., Wang S., Dong W., Quan W., Dang W., Yu X. (2017). Automatic schizophrenic discrimination on fNIRS by using complex brain network analysis and SVM. BMC Med. Inform. Decis. Mak..

[16] Garrity A.G., Pearlson G.D., McKiernan K., Lloyd D., Kiehl K.A., Calhoun V.D. (2007). Aberrant “Default Mode” Functional Connectivity in Schizophrenia. Am. J. Psychiatry.

[17] Woodward T.S., Leong K., Sanford N., Tipper C.M., Lavigne K.M. (2016). Altered balance of functional brain networks in Schizophrenia. Psychiatry Res. Neuroimaging.

[18] Sharma A., Weisbrod M., Kaiser S., Markela-Lerenc J., Bender S. (2010). Deficits in fronto-posterior interactions point to inefficient resource allocation in schizophrenia. Acta Psychiatr. Scand..

[19] Venkataraman A., Whitford T.J., Westin C.-F., Golland P., Kubicki M. (2012). Whole brain resting state functional connectivity abnormalities in schizophrenia. Schizophr. Res..

[20] Narayanaswamy J., Hazari N., Venkatasubramanian G. (2019). Neuroimaging findings in obsessive–compulsive disorder: A narrative review to elucidate neurobiological underpinnings. Indian J. Psychiatry.

[21] Fajnerova I., Gregus D., Francova A., Noskova E., Koprivova J., Stopkova P., Hlinka J., Horacek J. (2020). Functional Connectivity Changes in Obsessive–Compulsive Disorder Correspond to Interference Control and Obsessions Severity. Front. Neurol..

[22] Erdoğan S.B., Yükselen G., Yegül M.M., Usanmaz R., Kıran E., Derman O., Akın A. (2021). Identification of impulsive adolescents with a functional near infrared spectroscopy (fNIRS) based decision support system. J. Neural Eng..

[23] Çiftçi K., Sankur B., Kahya Y.P., Akin A. (2008). Multilevel Statistical Inference from Functional Near-Infrared Spectroscopy Data during Stroop Interference. IEEE Trans. Biomed. Eng..

[24] Aydore S., Mihcak M.K., Ciftci K., Akin A., Mihak M., Cifti K. (2009). On Temporal Connectivity of PFC via Gauss–Markov Modeling of fNIRS Signals. IEEE Trans. Biomed. Eng..

[25] Dadgostar M., Setarehdan S.K., Shahzadi S., Akin A. (2016). Functional connectivity of the PFC via partial correlation. Optik.

[26] Einalou Z., Maghooli K., Setarehdan S.K., Akin A. (2016). Effective channels in classification and functional connectivity pattern of prefrontal cortex by functional near infrared spectroscopy signals. Optik.

[27] Einalou Z., Maghooli K., Setarehdan S.K., Akin A. (2017). Graph theoretical approach to functional connectivity in prefrontal cortex via fNIRS. Neurophotonics.

[28] Akin A. (2017). Partial correlation-based functional connectivity analysis for functional near-infrared spectroscopy signals. J. Biomed. Opt..

[29] Akın A. (2021). fNIRS-derived neurocognitive ratio as a biomarker for neuropsychiatric diseases. Neurophotonics.

[30] Zysset S., Müller K., Lohmann G., von Cramon D. (2001). Color-Word Matching Stroop Task: Separating Interference and Response Conflict. Neuroimage.

[31] Erdogan S.B., Özsarfati E., Dilek B., Sogukkanlı Kadak K., Hanoglu L., Akin A. (2019). Classification of motor imagery and execution signals with population-level feature sets: Implications for probe design in fNIRS based BCI. J. Neural Eng..

[32] Akgül C.B., Akin A., Sankur B. (2006). Extraction of cognitive activity-related waveforms from functional near-infrared spectroscopy signals. Med. Biol. Eng. Comput..

[33] Erdoğan S.B., Yucel M., Akin A. (2014). Analysis of task-evoked systemic interference in fNIRS measurements: Insights from fMRI. Neuroimage.

[34] Pollonini L., Bortfeld H., Oghalai J.S. (2016). PHOEBE: A method for real time mapping of optodes-scalp coupling in functional near-infrared spectroscopy. Biomed. Opt. Express.

[35] Jobsis F.F. (1977). Noninvasive, infrared monitoring of cerebral and myocardial oxygen sufficiency and circulatory parameters. Science.

[36] Firbank M., Eiji O., Delpy D.T. (1998). A theoretical study of the signal contribution of regions of the adult head to near-infrared spectroscopy studies of visual evoked responses. Neuroimage.

[37] Cope M., Delpy D.T. (1988). System for long-term measurement of cerebral blood and tissue oxygenation on newborn infants by near infra-red transillumination. Med. Biol. Eng. Comput..

[38] Delpy D.T., Cope M., Zee P.V.D., Arridge S., Wray Sand Wyatt J. (1988). Estimation of optical pathlength through tissue from direct time of flight measurement. Phys. Med. Biol..

[39] Boas D.A., Dale A.M., Franceschini M.A. (2004). Diffuse optical imaging of brain activation: Approaches to optimizing image sensitivity, resolution, and accuracy. Neuroimage.

[40] Sutoko S., Monden Y., Tokuda T., Ikeda T., Nagashima M., Funane T., Atsumori H., Kiguchi M., Maki A., Yamagata T. (2020). Atypical dynamic-connectivity recruitment in attention-deficit/hyperactivity disorder children: An insight into task-based dynamic connectivity through an fNIRS study. Front. Hum. Neurosci..

[41] Al-Shargie F., Kiguchi M., Badruddin N., Dass S.C., Hani AF M., Tang T.B. (2016). Mental stress assessment using simultaneous measurement of EEG and fNIRS. Biomed. Opt. Express.

[42] Al-Shargie F., Tang T.B., Kiguchi M. (2017). Assessment of mental stress effects on prefrontal cortical activities using canonical correlation analysis: An fNIRS-EEG study. Biomed. Opt. Express.

[43] Hoshi Y. (2003). Functional near-infrared optical imaging: Utility and limitations in human brain mapping. Psychophysiology.

[44] Hoshi Y., Kobayashi N., Tamura M. (2001). Interpretation of near-infrared spectroscopy signals: A study with a newly developed perfused rat brain model. J. Appl. Physiol..

[45] Latora V., Marchiori M. (2001). Efficient behavior of small-world networks. Phys. Rev. Lett..

[46] Cui X., Bray S., Reiss A.L. (2010). Speeded near infrared spectroscopy (NIRS) response detection. PLoS ONE.

[47] Hawkins D.M., Basak S.C., Mills D. (2003). Assessing model fit by cross-validation. J. Chem. Inf. Comput. Sci..

[48] Tai K., Chau T. (2009). Single-trial classification of NIRS signals during emotional induction tasks: Towards a corporeal machine interface. J. Neuroeng. Rehabil..

[49] Sitaram R., Zhang H., Guan C., Thulasidas M., Hoshi Y., Ishikawa A., Shimizu K., Birbaumer N. (2007). Temporal classification of multichannel near-infrared spectroscopy signals of motor imagery for developing a brain–computer interface. Neuroimage.

[50] Tanaka H., Katura T. (2011). Classification of change detection and change blindness from near-infrared spectroscopy signals. J. Biomed. Opt..

[51] Misawa T., Takano S., Shimokawa T., Hirobayashi S. (2012). A brain-computer interface for motor assist by the prefrontal cortex. Electron. Commun. Jpn..

[52] Naseer N., Hong K.-S. (2015). fNIRS-based brain-computer interfaces: A review. Front. Hum. Neurosci..

[53] Naseer N., Qureshi N.K., Noori F.M., Hong K.-S. (2016). Analysis of Different Classification Techniques for Two-Class Functional Near-Infrared Spectroscopy-Based Brain-Computer Interface. Comput. Intell. Neurosci..

[54] Khan R.A., Naseer N., Qureshi N.K., Noori F.M., Nazeer H., Khan M.U. (2018). fNIRS-based Neurorobotic Interface for gait rehabilitation. J. Neuroeng. Rehabil..

[55] Shin J., Jeong J. (2014). Multiclass classification of hemodynamic responses for performance improvement of functional near-infrared spectroscopy-based brain-computer interface. J. Biomed. Opt..

[56] Shoushtarian M., Alizadehsani R., Khosravi A., Acevedo N., McKay C.M., Nahavandi S., Fallon J.B. (2020). Objective measurement of tinnitus using functional near-infrared spectroscopy and machine learning. PLoS ONE.

[57] Cearns M., Hahn T., Baune B.T. (2019). Recommendations and future directions for supervised machine learning in psychiatry. Transl. Psychiatry.

[58] Steardo L., Carbone E.A., De Filippis R., Pisanu C., Segura-Garcia C., Squassina A., De Fazio P. (2020). Application of Support Vector Machine on fMRI Data as Biomarkers in Schizophrenia Diagnosis: A Systematic Review. Front. Psychiatry.

[59] Yu Y., Shen H., Zeng L.-L., Ma Q., Hu D. (2013). Convergent and Divergent Functional Connectivity Patterns in Schizophrenia and Depression. PLoS ONE.

[60] Sakai K., Yamada K. (2018). Machine learning studies on major brain diseases: 5-year trends of 2014–2018. Jpn. J. Radiol..

[61] Arbabshirani M.R., Plis S., Sui J., Calhoun V.D. (2016). Single subject prediction of brain disorders in neuroimaging: Promises and pitfalls. Neuroimage.

[62] Stephan K.E., Schlagenhauf F., Huys Q., Raman S., Aponte E., Brodersen K., Rigoux L., Moran R., Daunizeau J., Dolan R. (2016). Computational neuroimaging strategies for single patient predictions. Neuroimage.

[63] Hall M., Frank E., Holmes G., Pfahringer B., Reutemann P., Witten I.H. (2009). The WEKA data mining software: An update SIGKDD. Explorations.

[64] Keerthi S.S., Shevade S.K., Bhattacharyya C., Murthy K.R.K. (2001). Improvements to Platt’s SMO algorithm for SVM classifier design. Neural Comput..

[65] Üstün B., Melssen W.J., Buydens L.M. (2006). Facilitating the application of support vector regression by using a universal Pearson VII function based kernel. Chemom. Intell. Lab. Syst..

[66] Zhang S., Yang G., Ou Y., Guo W., Peng Y., Hao K., Zhao J., Yang Y., Li W., Zhang Y. (2019). Abnormal default-mode network homogeneity and its correlations with neurocognitive deficits in drug-naive first-episode adolescent-onset schizophrenia. Schizophr. Res..

[67] Yamamuro K., Kimoto S., Iida J., Kishimoto N., Tanaka S., Toritsuka M., Ikawa D., Yamashita Y., Ota T., Makinodan M. (2018). Distinct patterns of blood oxygenation in the prefrontal cortex in clinical phenotypes of schizophrenia and bipolar disorder. J. Affect. Disord..

[68] Zhang Z., Wang Y., Zhang Q., Zhao W., Chen X., Zhai J., Chen M., Du B., Deng X., Ji F. (2019). The effects of CACNA1C gene polymorphism on prefrontal cortex in both schizophrenia patients and healthy controls. Schizophr. Res..

[69] Okada K., Ota T., Iida J., Kishimoto N., Kishimoto T. (2013). Lower prefrontal activity in adults with obsessive–compulsive disorder as measured by near-infrared spectroscopy. Prog. Neuro-Psychopharmacol. Biol. Psychiatry.

[70] Ota T., Iida J., Sawada M., Suehiro Y., Yamamuro K., Matsuura H., Tanaka S., Kishimoto N., Negoro H., Kishimoto T. (2012). Reduced Prefrontal Hemodynamic Response in Pediatric Obsessive–Compulsive Disorder as Measured by Near-Infrared Spectroscopy. Child Psychiatry Hum. Dev..

[71] Ahn S., Jun S.C. (2017). Multi-Modal Integration of EEG-fNIRS for Brain-Computer Interfaces—Current Limitations and Future Directions. Front. Hum. Neurosci..

[72] Batula A.M., Kim Y.E., Ayaz H. (2017). Virtual and Actual Humanoid Robot Control with Four-Class Motor-Imagery-Based Optical Brain-Computer Interface. BioMed Res. Int..

[73] Orru G., Pettersson-Yeo W., Marquand A.F., Sartori G., Mechelli A. (2012). Using support vector machine to identify imaging biomarkers of neurological and psychiatric disease: A critical review. Neurosci. Biobehav. Rev..

[74] Sen B., Bernstein G.A., Xu T., Mueller B.A., Schreiner M.W., Cullen K.R., Parhi K.K. Classification of obsessive-compulsive disorder from resting-state fMRI. Proceedings of the 2016 38th Annual International Conference of the IEEE Engineering in Medicine and Biology Society (EMBC).

[75] Chong C.D., Gaw N., Fu Y., Li J., Wu T., Schwedt T.J. (2016). Migraine classification using magnetic resonance imaging resting-state functional connectivity data. Cephalalgia.

[76] Yang H., Zhang J., Liu Q., Wang Y. (2018). Multimodal MRI-based classification of migraine: Using deep learning convolutional neural network. Biomed. Eng. Online.

[77] Jorge-Hernandez F., Chimeno Y.G., Garcia-Zapirain B., Zubizarreta A.C., Beldarrain M.A.G., Fernandez-Ruanova B. (2014). Graph theory for feature extraction and classification: A migraine pathology case study. Bio-Med. Mater. Eng..

[78] Kawasaki Y., Suzuki M., Kherif F., Takahashi T., Zhou S.-Y., Nakamura K., Matsui M., Sumiyoshi T., Seto H., Kurachi M. (2007). Multivariate voxel-based morphometry successfully differentiates schizophrenia patients from healthy controls. Neuroimage.

[79] Yassin W., Nakatani H., Zhu Y., Kojima M., Owada K., Kuwabara H., Gonoi W., Aoki Y., Takao H., Natsubori T. (2020). Machine-learning classification using neuroimaging data in schizophrenia, autism, ultra-high risk and first-episode psychosis. Transl. Psychiatry.

[80] Pardo P.J., Georgopoulos A.P., Kenny J.T., Stuve T.A., Findling R.L., Schulz S.C. (2006). Classification of adolescent psychotic disorders using linear discriminant analysis. Schizophr. Res..

[81] D’souza R.N., Huang P.Y., Yeh F.C. (2020). Structural Analysis and Optimization of Convolutional Neural Networks with a Small Sample Size. Sci. Rep..

[82] Wickramaratne S.D., Mahmud M.S. (2021). Conditional-GAN Based Data Augmentation for Deep Learning Task Classi-fier Improvement Using fNIRS Data. Front. Big Data.

[83] Khalil K., Asgher U., Ayaz Y. (2022). Novel fNIRS study on homogeneous symmetric feature-based transfer learning for brain-computer interface. Sci. Rep..

[84] Lyu B., Pham T., Blaney G., Haga Z., Sassaroli A., Fantini S., Aeron S. (2021). Domain adaptation for robust workload level alignment between sessions and subjects using fNIRS. J. Biomed. Opt..

[85] Hennrich J., Herff C., Heger D., Schultz T. Investigating Deep Learning for fNIRS Based BCI. Proceedings of the 2015 37th Annual International Conference of the IEEE Engineering in Medicine and Biology Society (EMBC).

[86] Chiarelli A.M., Croce P., Merla A., Zappasodi F. (2018). Deep Learning for Hybrid EEG-fNIRS Brain-Computer In-terface: Application to Motor Imagery Classification. J. Neural Eng..

